# Myelitis due to Coccidioidomycosis in an Immunocompetent Patient

**DOI:** 10.1155/2018/2176269

**Published:** 2018-04-01

**Authors:** Tetyana Vaysman, Sean Villaflores, Carlyn Estrella, Suman Radhakrishna, Antonio Liu

**Affiliations:** Department of Neurology and Infectious Disease, California Hospital Medical Center, Los Angeles, CA, USA

## Abstract

Myelitis of the spinal cord is an uncommon presentation of disseminated coccidioidomycosis. Most infected patients present subclinically, but patients, especially those who are immunocompromised, may progress to disseminated disease. We present a 50-year-old immunocompetent patient with no significant past medical history exhibiting symptoms of altered mental status, dizziness, headache, nausea, and quadriplegia. Upon investigation with lumbar puncture, cerebrospinal fluid (CSF) culture, and coccidioidal antibody studies, the patient was found to have acute coccidioidomycosis. Magnetic resonance imaging (MRI) of the brain demonstrated meningeal enhancements suggestive of meningitis, and further MRI study of the cervical spine revealed myelitis. Treatment with IV fluconazole for 2 weeks and IV voriconazole therapy over 3 weeks yielded limited improvement. The presentation of myelitis due to coccidioidomycosis infection is very rare and has infrequently reported in the literature. Awareness of this potentially fatal complication in immunocompetent patients can aid in faster recognition and treatment.

## 1. Introduction

Coccidioidomycosis, commonly referred to as valley fever, is caused by *Coccidioides immitis* and *Coccidioides posadasii*, two nearly identical species of pathogenic fungi most commonly found in southern Arizona, central California, southern New Mexico, and West Texas. California has a higher prevalence of *C. immitis* infection than *C. posadasii*. In immunocompetent persons, a self-limited acute pneumonia typically develops. Sixty percent of people infected with the fungus will remain asymptomatic or present with mild respiratory symptoms [1]. Approximately 40% of infections will lead to symptomatic disease, arising around one to four weeks after exposure, which can resemble influenza, with fever, cough, fatigue, dyspnea, headache, myalgia, and arthralgia [2]. A common initial presentation for a symptomatic patient is community acquired pneumonia [3]. Helpful diagnostic features include a history of night sweats, profound fatigue, peripheral blood eosinophilia, and hilar or mediastinal lymphadenopathy on chest radiography. A very low percentage of symptomatic infections will progress to the extrathoracic dissemination, with hematogenous spread to the central nervous system, skin, joints, major organs, or bones and result in chronic (months to years) and even fatal disease [4, 5]. Disseminated coccidioidomycosis may present in the central nervous system as abscesses, granulomas, or cystic lesions. The most common three subgroups of intracranial lesions were cranial osteomyelitis, leptomeningitis, and subcortical lesions [6]. Recently, both neuropathological and vasculopathic manifestations of the central nervous system coccidioidomycosis have been discussed [7]. Many cases with distinct CNS mass lesions and abscesses that were diagnosed with imaging techniques and confirmed by biopsy have been reported [8]. The term “myelitis” refers to inflammation of the spinal cord. The spectrum of neuropathological changes ranges from meningitis to meningoencephalitis and meningomyelitis with extensive parenchymal destruction, often as vasculitis and sometimes as a result of an associated endarteritis obliterans [7].

## 2. Case Report

**Figure 1:**
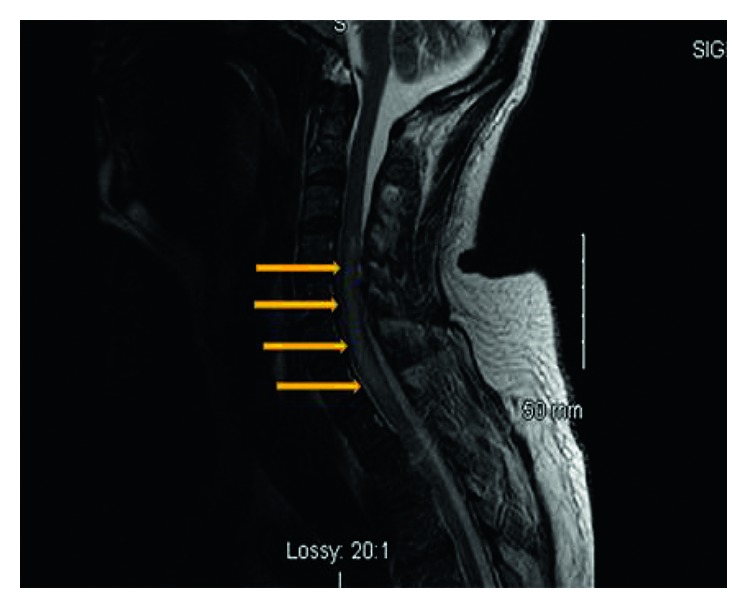
A sagittal T-weighted MRI of the C-spine of the patient on April 28, 2017, before the antifungal treatment. The arrows point to the spinal cord lesion due to coccidioidal infection.

A 50-year-old immunocompetent Hispanic male patient presented in April 2017 to the California Hospital Medical Center in Los Angeles with dizziness, nausea, headache, and a 2-week history of flu-like symptoms. Routine chest radiograph showed no abnormalities. Suspected bacterial meningitis was treated with vancomycin and ceftriaxone. Neurological examination revealed cranial nerves II through XII intact, no sensory or motor deficits, stable gait, DTRs 2+ bilaterally, and a negative Babinski sign. Serum lab results showed white blood cell count of 17,000/mm^3^. The patient had lumbar puncture done which revealed xanthochromic CSF fluid with a lymphocytic pleocytosis: WBC count of 234/mm^3^ (94% lymphocytes), glucose of 17 mg/dL, and CSF protein of 90 mg/dL. CT of the head showed ventricular enlargement and hydrocephalus with basal cistern patterns, but he did not require a shunt. Also, mild volume loss with small chronic ischemic disease was noted. Considering the CT of the head, dexamethasone was initiated, and the evaluation to establish the etiology of the meningitis was performed. Initial negative laboratory results were as follows: influenza A or B virus; CSF cryptococcal antigen; HIV-1/2 Ag/Ab screen; blood cultures; CSF cultures; and PCR for *Mycobacterium tuberculosis*. However, 6 days later the patient's condition worsened, and he was transferred to the ICU. The patient was not responding to the antibiotic and corticosteroid treatment, and his condition deteriorated rapidly over the next few days. *Coccidioidal meningitis* was confirmed with CSF *Coccidioides* complement fixing antibody titer of 1:2048. Treatment with IV fluconazole 1200 mg daily was then initiated. Neurological assessment demonstrated altered mental status; the patient was alert, oriented x1, responding to his name only, unable to follow commands, was able to move all extremities but had decreased grip strength bilaterally, held arms in flexion, and had difficulty feeding himself. Blunted left nasolabial fold was noted as a new manifestation. CT of the head demonstrated slightly improved hydrocephalus when compared to the previous exam. MRI of the brain showed meningeal enhancement in the basilar cisterns suggestive of basilar meningitis or a granulomatous process and 9 mm acute infarct in the right globus pallidus. Several days after initiation of fluconazole, follow-up neurological exam exhibited no improvement; the patient's motor strength gradually decreased with intact sensation and withdrawal to stimuli. MRI of C-spine (Figure 1) and T-spine without contrast revealed areas of signal changes in the cord secondary to myelitis and inflammation. MRI of L-spine without contrast showed crowding of the nerve roots in the lower lumbar spinal canal, suggestive of arachnoiditis, and explained the cause of the patient's significant neurological decline. The patient's motor strength decreased to 3/5 in proximal upper extremities and 0/5 in distal upper extremities with very poor hand grasp bilaterally. The patient lost bladder control, and his lower extremities were paralyzed completely even though his mentation improved.

## 3. Treatment

The drug of choice for the treatment of coccidioidal meningitis is fluconazole [9]. For patients who improve with therapy, this treatment has to be continued for life to prevent recurrence of the disease [10, 11]. In this particular case, the patient was placed on 1200 mg of IV fluconazole daily. However, 2 weeks after initiation of therapy, the patient showed improvement in mentation but exhibited progressive weakness in bilateral lower extremities and upper extremities. The treatment was escalated to IV voriconazole 400 mg twice daily, which is a recommended treatment for patients who do not respond to the initial treatment of choice [12, 13]. The patient was followed over another 4 weeks of treatment as inpatient. Some spontaneous movement in the lower extremities was observed despite the absence of voluntary control over the lower extremities. No changes in upper extremities and motor movement were noted even though the patient regained full mentation. Intrathecal amphotericin B treatment was considered due to the arrest in his condition with no significant improvement. Infectious diseases and neurosurgery specialists were consulted for placement of an Ommaya reservoir. Due to high incidence of coccidioidal infection in California, medical providers at the Kern Medical Center (Bakersfield, California) became the experts in providing treatment for coccidioidomycosis over 60 years. They report successful treatment and cure of the disease and reduction in mortality rates from 100% to 30% while using intrathecal amphotericin B deoxycolate for treatment of coccidioidal meningitis. They consider it as the only known option in the management of coccidioidal meningitis [14]. After explanation of all risks and benefits of the treatment with amphotericin B to the patient, continuous amphotericin B infusion via Ommaya reservoir was recommended by the medical team; however, the patient has declined the therapy. In this case, the patient's mental status has improved reflecting improvement in coccidioidal meningitis, but progressive myelitis of the spinal cord has resulted in quadriplegia. The treatment with intrathecal amphotericin B presents a high risk of arachnoiditis, which would not be an ideal option for this patient who has been already diagnosed with myelitis of the cervical spine and arachnoiditis of the lumbar spine. However, it would be a last option to change the course of the disease. The patient was followed with our inpatient services for 2 months and transferred as an outpatient on voriconazole. He was readmitted six months after discharge and remained quadriplegic with no neurological improvement. Five sets of MRI were done over two months, which showed the same results after initial improvement.

## 4. Discussion

Myelitis is a rare presentation of coccidioidomycosis. A case report published in 2016 described a 20-year-old healthy male with disseminated coccidioidomycosis and diffuse leptomeningeal enhancement, meningitis, and intramedullary spinal cord abscesses. The patient was treated with prolonged systemic liposomal amphotericin B, voriconazole, and an extended course of corticosteroids. Even though the patient was stabilized, he suffered severe neurological deficits along with the sequelae from the toxicity of steroids and antifungal treatment [15]. Rapidly progressing quadriparesis in the setting of disseminated coccidioidomycosis with cervical intramedullary involvement has been described in a case report of a 55-year-old man who required emergent cervical laminectomies with dural biopsy for decompression of the spinal cord and confirmation of the diagnosis and succumbed to the progressive course of the disease [16]. Disseminated coccidioidomycosis can mimic a lymphoma with bony metastases and thoracic myelopathy, as illustrated in the case report of a 23-year-old man who presented with respiratory distress and leg weakness [17]. In our case report, the disseminated coccidioidomycosis presented as new onset of myelitis. Interestingly, this is not a well-documented disease manifestation of coccidioidomycosis. Only a few cases of coccidioidal meningomyelitis have been described in literature, and it is extremely rare to see CNS myelitis due to coccidioidal infection in immunocompetent patients. It could be vividly seen that spinal cord involvement has limited prognostic features with poor neurologic and survival outcomes. The uniqueness of this case is that the neurological deficits have developed not due to the abscesses compressing the spinal cord but due to the inflammation of the spinal cord by itself resulting in nerve compression and subsequent neurological deficits.

**Figure 2:**
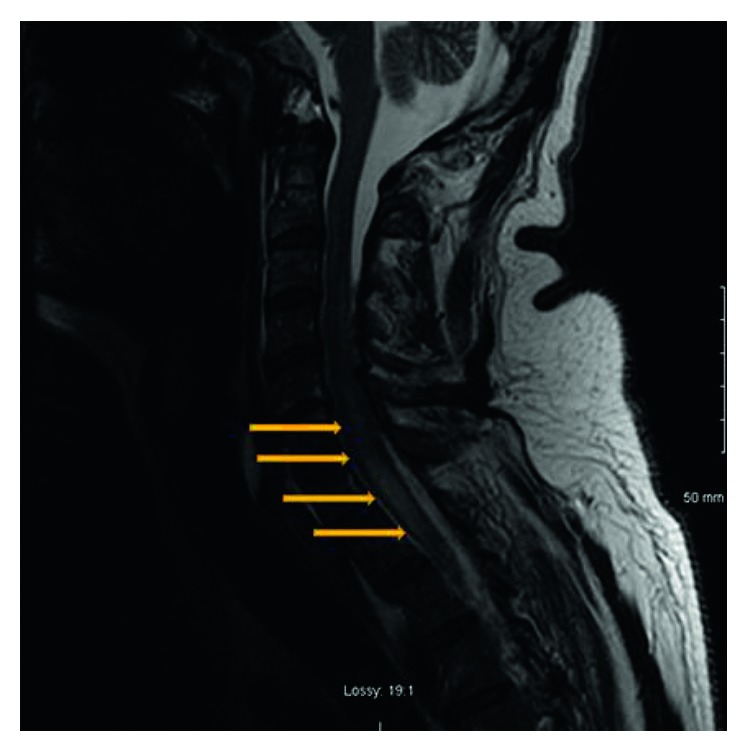
A sagittal T-weighted MRI of the C-spine of the patient on May 22, 2017, after the antifungal treatment. The arrows point to the spinal cord lesion due to coccidioidal infection; however, the size of the lesion has decreased significantly in comparison to Figure 1.

Myelitis is a very uncommon presentation of coccidioidomycosis (Figure 1). Initial treatment with IV fluconazole for 2 weeks resulted in significant improvement in mental status but no improvement in motor function of both the upper and lower extremities; IV voriconazole therapy over 3 weeks provided limited improvement in motor function and slightly decreased swelling of the spinal cord (Figure 2). The established treatment for coccidioidomycosis infection includes azole antifungals, such as fluconazole and itraconazole. Itraconazole is more efficacious for the treatment of skeletal disease but has poor CNS penetration. Posaconazole and voriconazole have also been used with some success in meningitis [18, 19, 20]. For patients with increased intracranial pressure at the time of meningitis diagnosis, repeated lumbar punctures are recommended [10]. Hydrocephalus may require VP shunting. Due to concern for possible spinal cord herniation in light of spinal cord edema, initial diagnostic lumbar punctures were performed, and any further lumbar punctures were deemed to be unsafe [21]. Along with the antifungal therapy, treatment with dexamethasone was provided to blunt spinal cord inflammation. In patients with CNS vasculitis with cerebrovascular accident due to coccidioidomycosis, those that received corticosteroids were less likely to develop additional infarcts compared to those who did not [22]. While intrathecal amphotericin B was previously the standard treatment, it is now reserved for the more severe or refractory cases or the first trimester of pregnancy [10]. However, intrathecal amphotericin B has very dangerous potential side effects such as arachnoiditis, vasculitis, and infarctions. To be effective, amphotericin B requires administration directly into the CSF [23, 24], either through an Ommaya reservoir or via lateral cervical puncture or lumbar route with the patient in head-down position. Treatment with intrathecal amphotericin may be necessary for years, adding additional risk and morbidity to its use. Considering the potential side effects in combination with the patient's current neurological status, the intrathecal treatment with amphotericin B could initially exacerbate his neurological deficits and lead to further neurologic deterioration. However, this is the most potent antifungal medication and the last resort to fight the infection. Amphotericin B was once the only effective treatment for coccidioidomycosis [25], and is still considered an effective, perhaps the most effective antifungal agent [26]. In collaboration with an infectious disease specialist and neurosurgical consultation, the intrathecal therapy was offered and recommended to the patient several times. All risks and benefits were discussed with the patient, who made the decision to decline the therapy due to potentially dangerous side effects of treatment. Future therapy will be determined based on the progression of the disease and its complications. Before the extensive use of CT and MRI imaging, the brain autopsy was the major diagnostic tool for disseminated coccidioidomycosis. With the availability of the noninvasive technology, we are able to both diagnose disseminated infection and monitor the progression of the disease. Any CNS coccidioidomycosis should warrant serial brain imaging, CSF antibody titer monitoring, and clinical re-evaluations. Since it will take months to years for improvement or recovery and because of high rate of recurrence, life-long azole therapy is indicated.

We hope our case study will raise awareness of prevalence and increased incidence of the coccidioidomycosis infection in California and facilitate prompt diagnosis in atypical patient presentation with similar symptoms. A high index of suspicion in such patients should guide rapid therapeutic management along with the different challenges encountered in care coordination. Aggressive approach is required to prevent further complications and neurological deficits.
